# 5-Aminolevulinic acid tumor paint and photodynamic therapy for myxofibrosarcoma: an in vitro study

**DOI:** 10.1186/s13018-020-01606-9

**Published:** 2020-03-05

**Authors:** Shachar Kenan, Haixiang Liang, Howard J. Goodman, Andrew J. Jacobs, Amanda Chan, Daniel A. Grande, Adam S. Levin

**Affiliations:** 1 Department of Orthopaedic Surgery, North Shore-Long Island Jewish Hospital, Northwell Health System, 270-05 76th Avenue, New Hyde Park, NY 11040 USA; 2 Orthopaedic Research Laboratory, The Feinstein Institute for Medical Research, 350 Community Drive, Manhasset, NY 11030 USA; 3 Hofstra Northwell School of Medicine, 500 Hofstra University, Hempstead, NY 11549 USA; 4 Microscopy Facility, The Feinstein Institute for Medical Research, 350 Community Drive, Manhasset, NY 11030 USA; 5 Department of Orthopaedic Surgery and Oncology, The Johns Hopkins University School of Medicine, 601 North Caroline Street, Baltimore, MD 21287 USA

**Keywords:** 5-Aminolevulinic acid, Myxofibrosarcoma, Neoadjuvant therapy, Photodynamic therapy, Sarcoma, Tumor paint

## Abstract

**Background:**

5-Aminolevulinic acid (5-ALA), a fluorescent contrast agent, has been used for tumor paint and photodynamic therapy (PDT) for various tumors, but its use with soft tissue sarcomas is not well documented. Myxofibrosarcoma, a subtype of soft tissue sarcoma with a high local recurrence rate, may benefit from similar types of treatment. The purpose of this study was to analyze the effects of 5-ALA tumor paint and PDT on a myxofibrosarcoma cell line.

**Methods:**

Tumor paint was assessed by exposing micromass pellets of human adipose-derived stromal (ADS) cells or myxofibrosarcoma (MUG-Myx1) cells to 5-ALA. Cell pellets were then visualized using a microscope at established excitation and emission wavelengths. Corrected total cell fluorescence was calculated per accepted protocols. Photodynamic therapy was similarly assessed by exposing ADS and MUG-Myx1 cells to 5-ALA, with subsequent analysis via flow cytometry and real-time confocal microscopy.

**Results:**

The use of 5-ALA tumor paint led to a selective fluorescence in MUG-Myx1 cells. Findings were confirmed by flow cytometry. Interestingly, flow cytometry results showed progressive selective cell death with increasing 5-ALA exposure as a result of the PDT effect. PDT was further confirmed using confocal microscopy, which revealed progressive cellular bubble formation consistent with advancing stages of cell death—a finding that was not seen in control ADS cells.

**Conclusions:**

5-ALA tumor paint and PDT were successfully used on a human myxofibrosarcoma cell line (MUG-Myx1). Results from this study showed both selective fluorescent tagging and selective cytotoxicity of 5-ALA toward malignant myxofibrosarcoma cells, while sparing benign adipose control cells. This finding was further confirmed in a dramatic time-lapse video, visually confirming active, targeted cell death. 5-ALA’s two-pronged application of selective tumor identification and cytotoxicity may transform surgical and medical approaches for treating soft tissue sarcomas.

## Background

Myxofibrosarcoma is a soft tissue sarcoma known for a high rate of local recurrence [1]. It is one of the most common sarcomas in elderly patients, mainly affecting patients during the sixth to eighth decades of life [2]. Myxofibrosarcoma typically occurs in the extremities and is less common in the trunk, neck, and feet. Although metastases are most commonly seen with intermediate- to high-grade tumors, recurrence rates have been reported to be 40 to 61% [1, 3], independent of tumor grade [4]. Treatment typically involves a combination of surgery, radiation, and chemotherapy.

Curative treatment for extremity soft tissue sarcomas requires resection with wide margins. Despite frequently being located superficial to the fascia, myxofibrosarcoma tends to spread along the fascial planes with associated large areas of peritumoral edema. It has been shown that this reactive zone of edema contains sarcoma cells [5]. Because it is difficult to identify the extent of microscopic fascial spread, surgical treatment may require multiple re-excisions to obtain microscopically negative margins. Preoperative magnetic resonance imaging can aid in surgical planning, but tumor size is often underestimated [6].

Intraoperative assessment of tumor margins is difficult. “Tumor paint” is an optical imaging contrast agent used intraoperatively to help differentiate cancer foci from adjacent healthy tissues [7]. The concept of fluorescence-guided resection using tumor paint to differentiate a tumor from adjacent normal tissue was pioneered by Stummer et al. [8] in glioblastoma resection.

Photodynamic therapy (PDT) uses a photosensitizer drug that is activated by a light source to exert cytotoxic activity toward cells [9]. In 1987, a breakthrough in the field of PDT was made when Malik and Lugaci [10] described 5-aminolevulinic acid (5-ALA), which became the first second-generation photosensitizer. Unlike earlier photosensitizers, 5-ALA is an inactive precursor in the heme pathway; therefore, the risk of adverse phototoxic effects that plagued its predecessors was reduced significantly. 5-ALA is ultimately converted to the active photosensitizer, protoporphyrin IX, within the mitochondria. Because of abnormal accumulation of this photosensitizer, final downstream effects are specifically targeted to pathologic cells, rather than normal cells.

Exogenous 5-ALA has been approved by the United States Food and Drug Administration (FDA) for topical and oral use [11, 12]. 5-ALA is a safe fluorescent contrast agent that has been used for PDT and intraoperative margin identification of various types of tumors, but its use with soft tissue sarcomas is poorly documented. The purpose of this study was to assess the in vitro applications of 5-ALA tumor paint and PDT on myxofibrosarcoma (MUG-Myx1) cells. Our first hypothesis was that MUG-Myx1 cells would fluoresce more strongly than control human adipose-derived stromal (ADS) cells in response to 5-ALA, allowing for visual differentiation of malignant cells from benign cells. Our second hypothesis was that 5-ALA PDT would selectively induce cell death in MUG-Myx1 cells while sparing benign adipose cells.

## Methods

Tumor paint and PDT represented the two arms of this study. The following two cell lines were used for both: normal human ADS cells (Lonza Inc., Allendale, NJ) and a human MUG-Myx1 myxofibrosarcoma cell line, recently authenticated by STR profiling and tested negative for mycoplasma contamination (University of Graz, Graz, Austria) [13]. All cells remained free of contaminants and were propagated according to established protocols [13, 14]. Cells were cultured using Dulbecco’s modified Eagle/F12 (50:50) medium (DMEM/F12) (Corning Life Sciences, Teterboro, NJ) containing 10% fetal bovine serum (FBS), 1% l-glutamine, 100 units/mL of penicillin, 100 μg/mL of streptomycin, and 0.25 μg/mL of amphotericin B. The medium was changed every 2 days. Cells were maintained at 37 °C in 5% CO_2_.

### Tumor paint

ADS cells and human MUG-Myx1 cells were cultured separately to form discrete micromass cell pellets using hanging drop techniques [15]. Each micromass consisted of a 3-dimensional sphere of 5000 cells bathed in DMEM/F12 medium with 10% FBS. After 24 h, the pellets were collected and cultured in a 96-well round-bottom low attachment plate (Corning Life Sciences, Teterboro, NJ). All micromasses, other than the control group, were treated with 5-ALA at a concentration of 500 μg/mL for 3 h. After 3 h, the cell pellets were split into two groups: one bathed in the 5-ALA medium and the other for which the medium was washed and replaced with normal, 5-ALA-free medium. This resulted in three distinct experimental groups as follows: untreated control, 5-ALA removed, and 5-ALA treated. There were two samples per group.

#### Microscope parameters

Cell pellets were visualized at × 100 magnification using a wide-field inverted fluorescence microscope (Zeiss Axiovert 200M, Carl Zeiss Microscopy, LLC, Peabody, MA) 20 min after the medium exchange. 5-ALA fluorescence was visualized using a Zeiss filter with an excitation bandpass range of 395–440 nm and a long pass 470-nm emission (Carl Zeiss Microscopy, LLC). Except for the time needed to handle the cells under visible light in a laboratory hood, all cell pellets were kept in insulated chambers, protected from exposure to outside light, before being observed under the microscope.

#### Image analysis

Corrected total cell fluorescence (CTCF) was calculated using the ImageJ software [16], version 1.48 [17], according to previously described protocols to control for local background fluorescence and cell size. The following formula was used [16]:

CTCF = integrated density − (area of selected cell × mean fluorescence of background readings).

Positive CTCF scores indicate cells that fluoresce relative to their local backgrounds. Negative CTCF scores indicate cells that appear darker than their local backgrounds. A red filter was applied for contrast, and final images were saved in a tagged image file format (TIFF).

#### Statistics

Statistical analysis was performed using Microsoft Excel. Paired *t* tests (*α* = 0.05) were performed to assess the statistical significance when comparing ADS cell fluorescence versus MUG-Myx1 fluorescence using their respective CTCF values.

### PDT

MUG-Myx1 and ADS cells were treated with 5-ALA at a concentration of 500 μg/mL when they had both reached 70% confluence. After 1 or 3 h, cells were lifted with 0.05% trypsin in ethylenediaminetetraacetic acid (EDTA) and washed twice with phosphate-buffered saline (PBS). Cells were then suspended in PBS with 0.1% bovine serum albumin for the flow cytometry and confocal microscopy studies.

#### Flow cytometry

Flow cytometry using a BD LSRFortessa X-20 cell analyzer (BD Biosciences, Sparks, MD) was performed 20 min or 1 h after the medium exchange. There were three resulting groups per cell line. Group 1 was analyzed 20 min after medium exchange following 1 h of 5-ALA exposure, group 2 was analyzed 20 min after medium exchange following 3 h of 5-ALA exposure, and group 3 was analyzed 1 h after medium exchange following 3 h of 5-ALA exposure.

#### Confocal microscopy PDT

MUG-Myx1 cells that had been exposed to 3 h of 5-ALA were visualized using a confocal laser scanning microscope (LSM 880 with Airyscan, Carl Zeiss Microscopy, LLC) with a × 10 objective at 405-nm excitation and emission at a 603–738-nm wavelength. Time-lapse images of live cells were captured using bright field and 405-nm laser every second for 20 min.

## Results

### Tumor paint

**Fig. 1:**
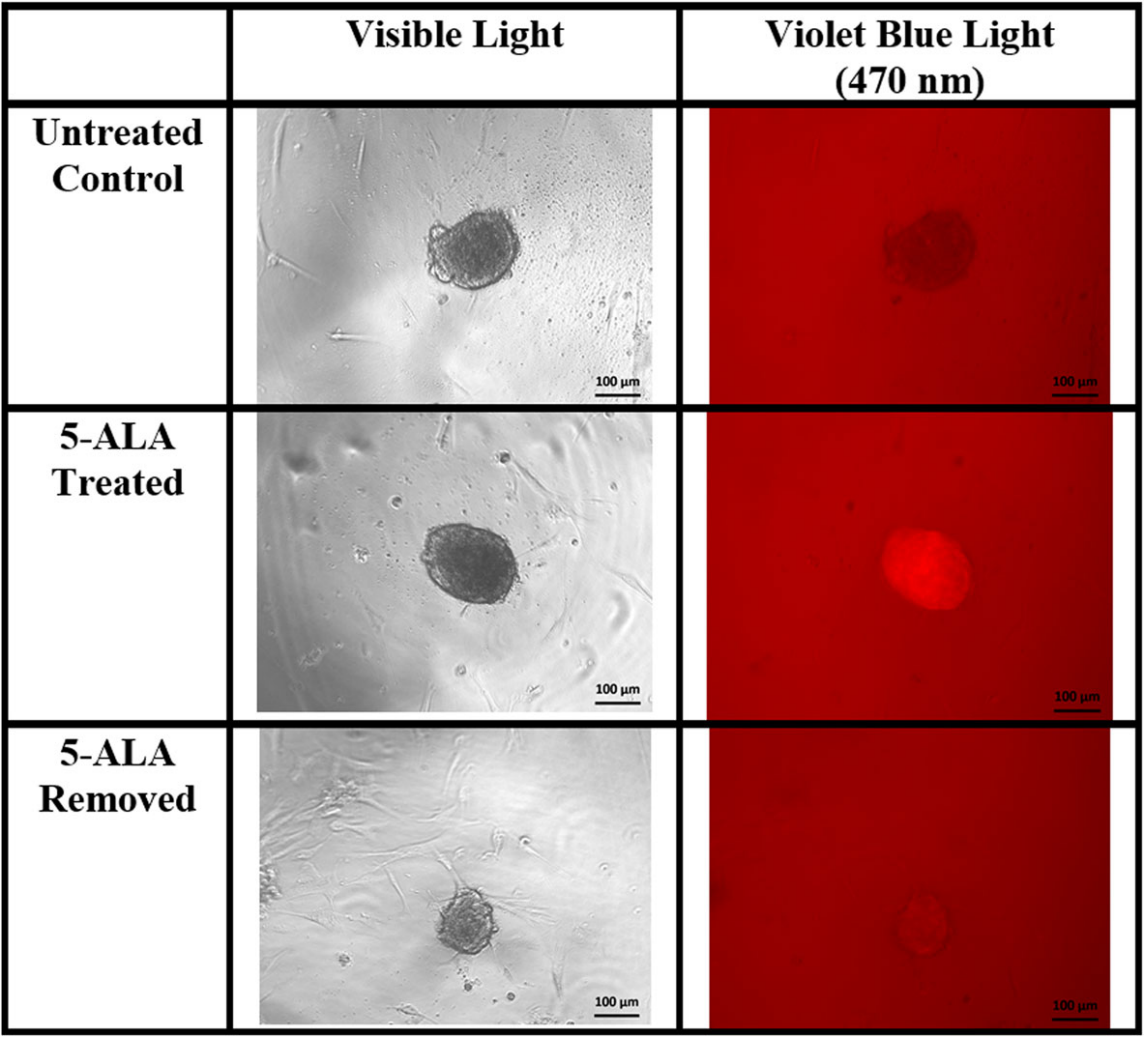
Human adipose-derived stromal (benign) micromass images, × 100 magnification. Fluorescence nearly eliminated when 5-aminolevulinic acid (5-ALA) medium is removed (lower right image)

**Fig. 2:**
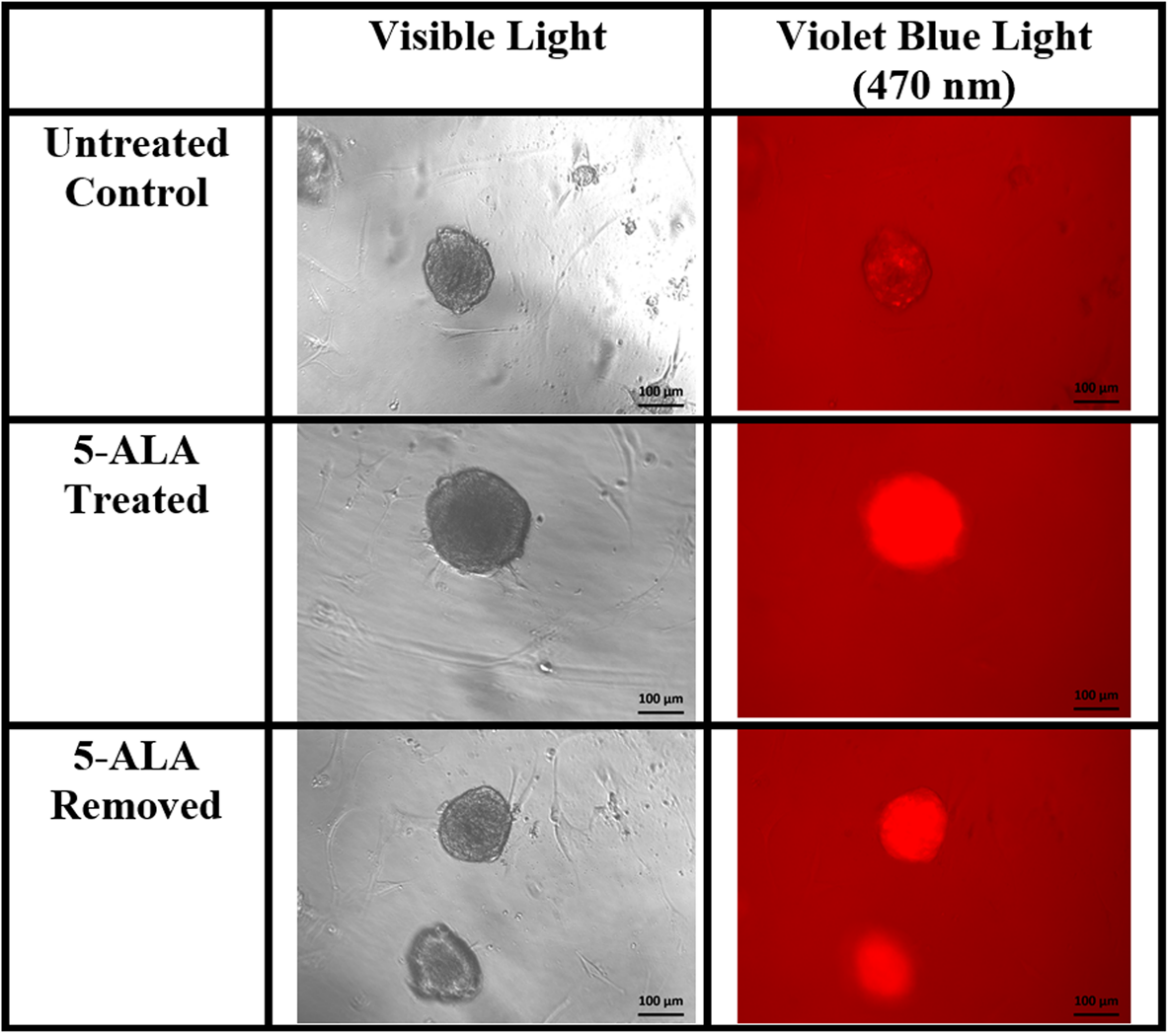
Myxofibrosarcoma (malignant) micromass images, × 100 magnification. Fluorescence persists when 5-aminolevulinic acid (5-ALA) medium is removed (lower right image)

The mean CTCF values for the ADS cell line in the untreated control group, the 5-ALA removed group, and the 5-ALA treated groups were − 1,543,430 units (standard deviation (SD), 4106), 87,454 units (SD, 41,005), and 813,063 units (SD, 79,187), respectively (Fig. 1). The mean CTCF values for the MUG-Myx1 cell line in the untreated control group, the 5-ALA removed group, and the 5-ALA treated groups were 53,266 units (SD, 31,175), 2,748,070 units (SD, 395,211), and 4,630,604 units (SD, 1,040,675), respectively (Fig. 2).

**Fig. 3:**
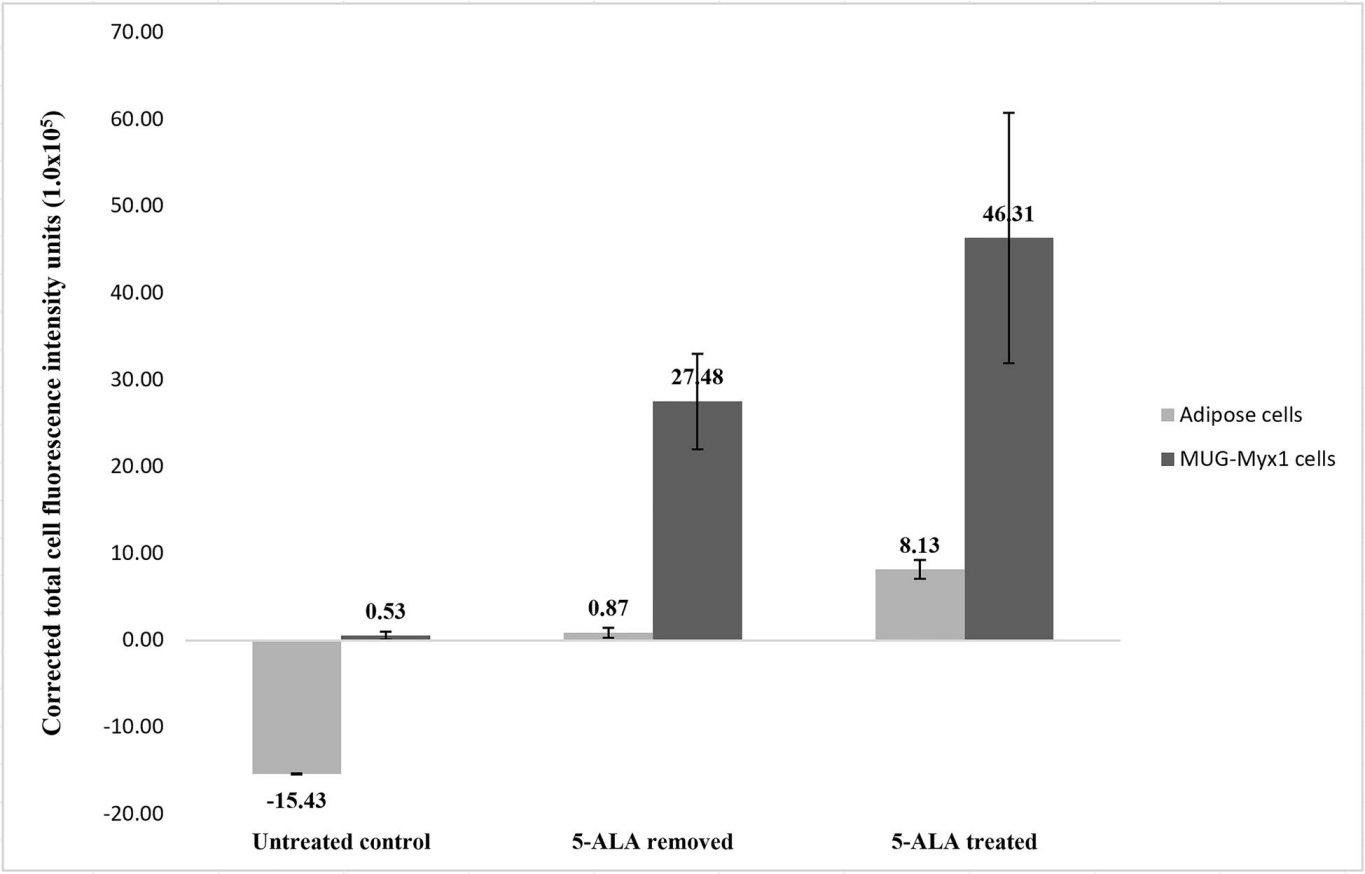
Corrected total cell fluorescence of human adipose-derived stromal and myxofibrosarcoma cells. 5-ALA, 5-aminolevulinic acid; MUG-Myx1, myxofibrosarcoma

The MUG-Myx1 cell pellets had higher CTCF values than ADS cell pellets in all three groups; however, this difference was significant in only the control and 5-ALA removed groups (Fig. 3; *p* < 0.05). The ADS and MUG-Myx1 cell pellets had the highest CTCF values after the addition of 5-ALA without a medium exchange. Interestingly, fluorescence persisted after 5-ALA removal in the MUG-Myx1 cell pellets but not in the ADS cell pellets, where fluorescence quickly diminished to baseline levels.

#### Flow cytometry

**Fig. 4:**
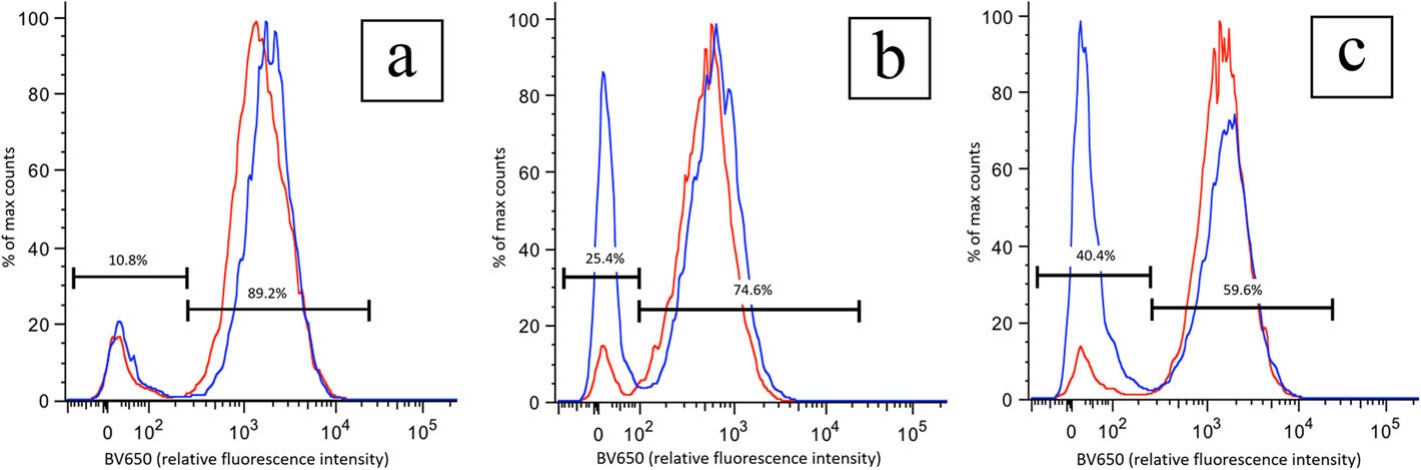
Flow cytometry fluorescence intensity graphs. **a** Group 1, 20 min after the medium exchange with 1 h of 5-aminolevulinic acid (5-ALA) exposure. High peak represents viable cells, accounting for 89% of myxofibrosarcoma (MUG-Myx1) cells (blue). Low peak represents unresponsive or dead cells, accounting for 11% of MUG-Myx1 cells. **b** Group 2, 20 min after the medium exchange with 3 h of 5-ALA exposure. High peak drops to 76% of MUG-Myx1 cells. Low peak increases to 25% of MUG-Myx1 cells. **c** Group 3, 1 h after the medium exchange with 3 h of 5-ALA exposure. High peak drops to 60% of MUG-Myx1 cells. Low peak increases to 40% of MUG-Myx1 cells. Human adipose-derived stromal cell peaks (red) remain unchanged

The ADS and MUG-Myx1 cell lines displayed two isolated peaks of fluorescence for each group. The first peak (closer to the origin point) represents cells that have not responded to 5-ALA. The second peak represents 5-ALA-responsive cells. The first group had an ADS low peak of 34 intensity units and a high peak of 1889 intensity units (Fig. 4a). At this same time point, the MUG-Myx1 peaks were similar to the ADS peaks, although slightly higher, at 43 and 2198 intensity units, respectively. Cell distributions were similar in both groups, with approximately 10% of cells representing the low peak and 90% of cells representing the high peak.

The MUG-Myx1 cell distributions differed for the second and third groups, with low peak distributions increasing from 11 to 25% and 40%, respectively, and high peak distributions decreasing from 89 to 75% and 60%, respectively (Fig. 4b, c). This was unlike the ADS cell line, in which the cell distribution remained unchanged regardless of the length of exposure to 5-ALA or time after the medium exchange.

#### Confocal microscopy PDT

**Fig. 5:**
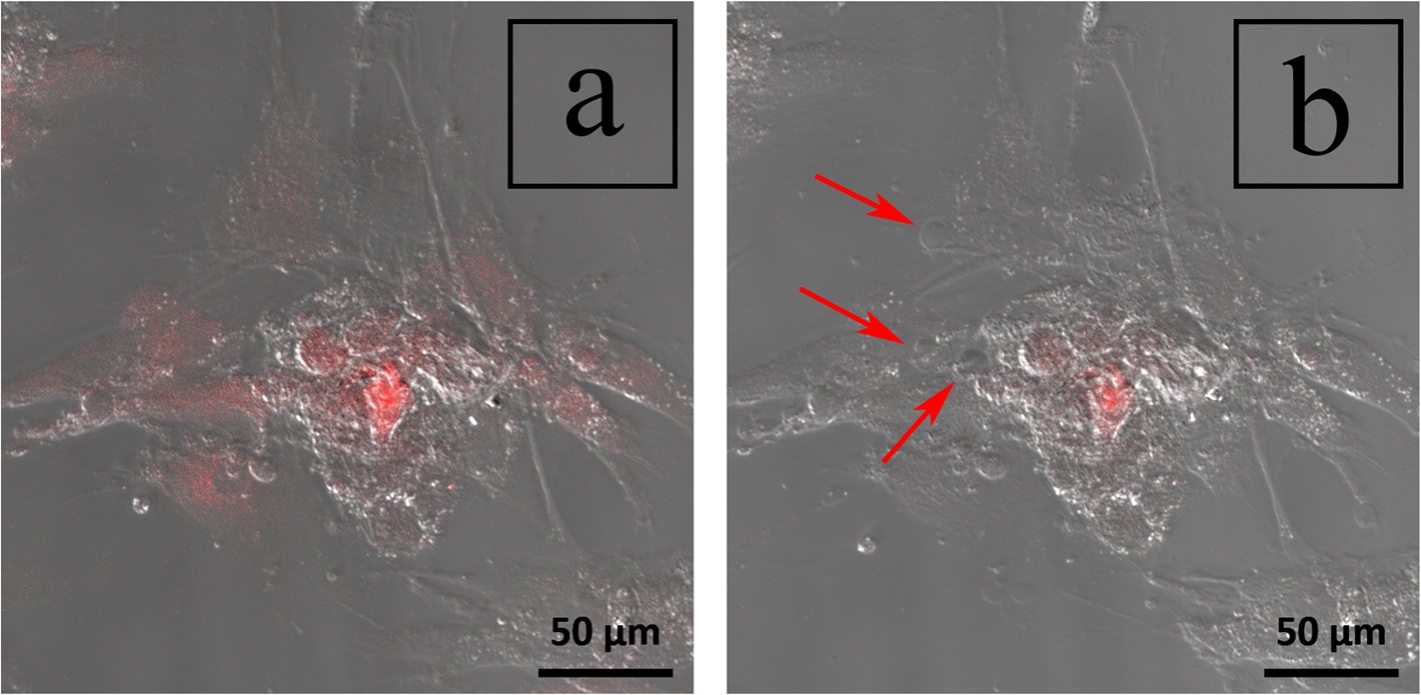
Confocal microscopy photodynamic therapy using a 405-nm laser at **a** time 0 and **b** 20 min after exposure. Red arrows show numerous rapidly enlarging vesicles indicating rapidly progressing cell death

At the start of this experiment, the affected myxofibrosarcoma cells can be seen fluorescing red at an emission wavelength of 602–738 nm in response to the 405-nm excitation laser exposure (Fig. 5a). The cells appear myxoid with fibrous tentacle-like villous structures, typical of myxofibrosarcoma cells. The 1200 captured frames were then visualized sequentially at 50× speed, resulting in a 7-s video (Additional file 1). As time progressed, there appeared to be increased intracellular swelling, with a rapid formation of multiple vesicles exiting from the cellular membrane (Fig. 5b). The ADS cells, in contrast, were unaffected, showing no signs of vesicle formation when exposed to similar conditions (Additional file 2).

## Discussion

Our primary goals were to assess (1) the ability of 5-ALA to fluoresce MUG-Myx1 cells preferentially under violet-blue light and (2) the selective cytotoxic effects of 5-ALA on MUG-Myx1 cells versus nonmalignant control cells using flow cytometry and confocal microscopy PDT. We found that 5-ALA combined with exposure to violet-blue light led to selective MUG-Myx1 tumor cell fluorescence and cell death while sparing nonmalignant control cells.

Micromasses, 3-dimensional spheroid models, were chosen for this experiment because of their proven effectiveness for the development and testing of various “anticancer photodynamic therapy regimens” [18]. In our study, MUG-Myx1 cells, when present as a micromass in vitro, demonstrated an increased level of fluorescence after 5-ALA treatment. Additionally, the time period during which tumor cells maintained a higher level of fluorescence was longer than that of normal ADS cells after the removal of 5-ALA. This discovery suggests that a single-dose administration of 5-ALA followed by the elimination of the drug by the body will help distinguish tumor cells from surrounding normal tissue.

Recently, 5-ALA has shown promising results in various disciplines, including neurosurgery [8, 19–22], urology [23–25], gynecology [14, 26, 27], and dermatology [28–30]. Denzinger et al. [23] found a significant drop in residual tumor rate in 88 patients treated with 5-ALA-assisted cystoscopy (4.5%), as opposed to the 103 patients without (25%) (*p* = 0.0003). A meta-analysis of 10 studies involving intraoperative 5-ALA fluorescence-guided resections of gliomas suggested that 5-ALA-guided surgery is more effective than conventional surgery, enhancing the quality of life and prolonging survival [31]. A survival analysis of 646 patients across 3 studies was performed as part of this meta-analysis, with a 41 to 46% progression-free survival rate using 5-ALA-guided resections, as opposed to 21 to 28% for resections under plain visible light [31].

In the field of orthopedics, Bickels [32] is leading an ongoing phase 2 single-center trial using 5-ALA and a red light laser (635 nm) as a means of tumor bed phototherapy after desmoid tumor resection. In another study [33], the same group cited 24 patients with fibrotic soft tissue tumors, including 12 desmoid and 3 each of fibrosarcoma, dermatofibrosarcoma protuberans, solitary fibrous tumors, and myxofibrosarcoma. All tumor beds were treated with 635-nm red light laser 5-ALA PDT after excision. There were 4 local recurrences, all of which occurred in patients with desmoid tumors, 3 of which were possibly not exposed to sufficient light. Although this study did not use 5-ALA to guide resection, it did report no added morbidity associated with the treatment [33]. Two other reports cited 5-ALA and its application for PDT in osteosarcoma [34, 35]. Li et al. [34] showed a significant inhibition of osteosarcoma growth in an in vivo rat model; however, this inhibition was related to 5-ALA sonodynamic therapy using low-intensity ultrasonography rather than light to trigger the apoptotic effects. The in vitro study by White et al. [35] analyzed human osteosarcoma cells treated with 5-ALA exposed to either 40-nM excitation wavelength light or kept in the dark for 24 h. Their results showed maximal human osteosarcoma cell death in the light-exposed group with approximately 60–70% cell viability as opposed to more than 90% viability in the dark group. Finally, consistent with the current study results, findings from a more recent report on a variety of carcinomas and sarcomas demonstrated in vitro cytotoxicity and fluorescence in response to 5-ALA application; however, this was noted in veterinary tumors, none of which included myxofibrosarcoma [36]. This veterinary study reported a sensitivity of 89% and a specificity of 50% for red fluorescence across 144 resected tumors. Furthermore, 5-ALA PDT was assessed in vivo in 10 dogs and 4 cats using a 630-nm wavelength. At a mean follow-up of 184 days, the response was assessed using RECIST criteria, with 2 of 14 showing a complete response, 6 showing a partial response, 3 showing a stable disease, and 3 showing a disease progression.

The fluorescence induced by the treatment with 5-ALA was confirmed by flow cytometry. Longer treatment time and longer elapsed time after treatment led to reduced fluorescence in tumor cells. We consider this reduction in fluorescence to represent cellular death. In contrast, control ADS cells had no difference in fluorescence under these same conditions. This result indicates that the cytotoxicity is selective to tumor cells after treatment with 5-ALA. 5-ALA has a favorable toxicity profile, with the most common adverse effects being mild alterations of blood cell counts without clinical symptoms and slight increases in liver enzymes [37]. There is little evidence to support symptoms or laboratory results of clinical relevance [38]. With an array of clinical applications in various tissues, 5-ALA has been approved by the FDA for intravesical instillation for detecting bladder cancer during cystoscopy and for topical application for PDT to treat basal cell carcinoma and actinic keratosis [11]. It was approved for the treatment of gliomas by the European Medicines Agency in 2007 and, as of June 2017, has been approved by the FDA as an orally administered optical contrast agent in patients with gliomas “as an adjunct for the visualization of malignant tissue during surgery” [12, 39].

In the current study, we found that the cytotoxicity of 5-ALA to the tumor cells was related to the length of 5-ALA exposure. The cytotoxic effect was also found to persist after the removal of the 5-ALA, indicating this effect was caused by a triggered intracellular cascade of events rather than a direct killing by 5-ALA itself. Of note, the cells were exposed to a limited amount of room light during a short time when preparing for the flow cytometry study. The cytotoxic effect on tumor cells could therefore be considered light-independent.

5-ALA-driven PDT was confirmed by the laser treatment under confocal microscopy. The bubble forming on the membrane of the cells indicated bubbling cell death, which is considered evidence of DNA damage caused by the accumulation of nitric oxide [40]. It has been reported that the PDT induced by 5-ALA exerts cytotoxic effects on malignant cells by activating the photosensitizer protoporphyrin IX. Type 1 reactions, which release free radicals, and type 2 reactions, which release singlet oxygen species, ultimately lead to advancing stages of cell death, including chromatin aggregation, mitochondrial damage, water influx, and lysis of the cytosol [9, 10]. In their landmark 1987 study of 5-ALA, Malik and Lugaci [10] noted a 95% erythroleukemic cell destruction in 5-ALA-treated cells that were exposed to 6 days of “black-light” (320 to 450 nm); cells that were not exposed to light survived. Our study confirmed the PDT effect on myxofibrosarcoma cells induced by 5-ALA treatment.

## Conclusions

This in vitro study shows 5-ALA’s selectivity for MUG-Myx1 cells and its relative resistance to normal adipose tissue. The tumor paint results show the capacity of MUG-Myx1 cells to retain fluorescence in the absence of continuous 5-ALA exposure. Clinically, this suggests that cells may continue to fluoresce intraoperatively in a dynamic environment such as the tumor bed, where arterial and venous blood flow, as well as intraoperative irrigation, may wash away the 5-ALA. The PDT time-lapse video dramatically reveals the cytotoxic effects of 5-ALA on MUG-Myx1 cells, a display that, to the author’s knowledge, has not been described elsewhere. These findings have the potential to make a major clinical impact on the way soft tissue sarcomas are treated surgically through improved margin identification, decreased recurrence rates, and minimized surgical site morbidity.

## Supplementary information

**Additional file 1.** Time-lapse video of MUG-Myx1 cells after exposure to 5-ALA PDT. Description: Time-lapse video showing photodynamic therapy using a 405-nm laser with confocal microscopy to target myxofibrosarcoma cells. Video speed is 50× real time, accounting for a 20-minute period of time. Multiple intracellular vesicles can be seen rapidly enlarging, indicating initial stages of cell death.

**Additional file 2.** Time-lapse video of ADS cells after exposure to 5-ALA PDT. Description: Time-lapse video showing attempted photodynamic therapy using a 405-nm laser with confocal microscopy to target adipose derived stromal cells. Video speed is 50× real time, accounting for a 20-minute period of time. No remarkable changes are appreciated after treatment.

## Data Availability

All datasets on which the conclusions of this report rely are available on request.

## Abbreviations

5-ALA: 5-Aminolevulinic acid
ADS: Adipose-derived stromal cell line
CTCF: Corrected total cell fluorescence
DMEM/F12: Dulbecco’s modified Eagle/F12
FBS: Fetal bovine serum
FDA: Food and Drug Administration
MUG-Myx1: Myxofibrosarcoma cell line
PBS: Phosphate-buffered saline
PDT: Photodynamic therapy
SD: Standard deviation
TIFF: Tagged image file format

## References

[1] Weiss SW, Enzinger FM (1977). Myxoid variant of malignant fibrous histiocytoma. Cancer..

[2] Fletcher CDM, Unni KK, Mertens F (2002). Pathology & genetics: tumours of soft tissue and bone.

[3] Haglund KE, Raut CP, Nascimento AF, Wang Q, George S, Baldini EH (2012). Recurrence patterns and survival for patients with intermediate- and high-grade myxofibrosarcoma. Int J Radiat Oncol Biol Phys..

[4] Mentzel T, Calonje E, Wadden C (1996). Myxofibrosarcoma: clinicopathologic analysis of 75 cases with emphasis on the low-grade variant. Am J Surg Pathol..

[5] Manoso MW, Pratt J, Healey JH, Boland PJ, Athanasian EA (2006). Infiltrative MRI pattern and incomplete initial surgery compromise local control of myxofibrosarcoma. Clin Orthop Relat Res..

[6] Riouallon G, Larousserie F, Pluot E, Anract P (2013). Superficial myxofibrosarcoma: assessment of recurrence risk according to the surgical margin following resection. A series of 21 patients. Orthop Traumatol Surg Res..

[7] Veiseh M, Gabikian P, Bahrami SB (2007). Tumor paint: a chlorotoxin:Cy5.5 bioconjugate for intraoperative visualization of cancer foci. Cancer Res..

[8] Stummer W, Novotny A, Stepp H, Goetz C, Bise K, Reulen HJ (2000). Fluorescence-guided resection of glioblastoma multiforme by using 5-aminolevulinic acid-induced porphyrins: a prospective study in 52 consecutive patients. J Neurosurg..

[9] Wachowska M, Muchowicz A, Firczuk M (2011). Aminolevulinic acid (ALA) as a prodrug in photodynamic therapy of cancer. Molecules..

[10] Malik Z, Lugaci H (1987). Destruction of erythroleukaemic cells by photoactivation of endogenous porphyrins. Br J Cancer..

[11] Krammer B, Plaetzer K (2008). ALA and its clinical impact, from bench to bedside. Photochem Photobiol Sci..

[12] U.S. Food and Drug Administration. Aminolevulinic acid hydrochloride, known as ALA HCl (Gleolan, NX Development Corp.) as an optical imaging agent indicated in patients with gliomas. Available at https://www.fda.gov/Drugs/InformationOnDrugs/ApprovedDrugs/ucm562645.htm. Accessed on May 4, 2018.

[13] Lohberger B, Stuendl N, Wolf E, Liegl-Atzwanger B, Leithner A, Rinner B (2013). The novel myxofibrosarcoma cell line MUG-Myx1 expresses a tumourigenic stem-like cell population with high aldehyde dehydrogenase 1 activity. BMC Cancer..

[14] Millon SR, Ostrander JH, Yazdanfar S (2010). Preferential accumulation of 5-aminolevulinic acid-induced protoporphyrin IX in breast cancer: a comprehensive study on six breast cell lines with varying phenotypes. J Biomed Opt..

[15] Aijian AP, Garrell RL (2015). Digital microfluidics for automated hanging drop cell spheroid culture. J Lab Autom..

[16] Fitzpatrick M. Measuring cell fluorescence using ImageJ. Available at http://theolb.readthedocs.io/en/latest/imaging/measuring-cell-fluorescence-using-imagej.html#measuring-cell-fluorescence-using-imagej. Accessed on August 4. 2017.

[17] Rasband W. ImageJ image processing and analysis in Java. Available at http://rsb.info.nih.gov/ij/index.html. Accessed on June 30, 2015.

[18] Evans CL. Three-dimensional in vitro cancer spheroid models for photodynamic therapy: strengths and opportunities. Available at https://www.frontiersin.org/articles/10.3389/fphy.2015.00015/full. Accessed on May 4, 2018.

[19] Goryaynov SA, Okhlopkov VA, Golbin DA (2019). Fluorescence diagnosis in neurooncology: retrospective analysis of 653 cases. Front Oncol..

[20] Picart T, Berhouma M, Dumot C (2019). Optimization of high-grade glioma resection using 5-ALA fluorescence-guided surgery: a literature review and practical recommendations from the neuro-oncology club of the French society of neurosurgery. Neurochirurgie..

[21] Schwake M, Schipmann S, Muther M, Kochling M, Brentrup A, Stummer W (2019). 5-ALA fluorescence-guided surgery in pediatric brain tumors-a systematic review. Acta Neurochir (Wien).

[22] Stummer W, Stepp H, Wiestler OD, Pichlmeier U (2017). Randomized, Prospective double-blinded study comparing 3 different doses of 5-aminolevulinic acid for fluorescence-guided resections of malignant gliomas. Neurosurgery..

[23] Denzinger S, Burger M, Walter B (2007). Clinically relevant reduction in risk of recurrence of superficial bladder cancer using 5-aminolevulinic acid-induced fluorescence diagnosis: 8-year results of prospective randomized study. Urology..

[24] Denzinger S, Rossler W, Otto W (2007). Photodynamic diagnostic of superficial bladder carcinoma. Dtsch Med Wochenschr..

[25] Regula J, MacRobert AJ, Gorchein A (1995). Photosensitisation and photodynamic therapy of oesophageal, duodenal, and colorectal tumours using 5 aminolaevulinic acid induced protoporphyrin IX--a pilot study. Gut..

[26] Matoba Y, Banno K, Kisu I, Aoki D (2018). Clinical application of photodynamic diagnosis and photodynamic therapy for gynecologic malignant diseases: a review. Photodiagnosis Photodyn Ther..

[27] Teshigawara T, Mizuno M, Ishii T (2018). Novel potential photodynamic therapy strategy using 5-aminolevulinic acid for ovarian clear-cell carcinoma. Photodiagnosis Photodyn Ther..

[28] Fu C, Kuang BH, Qin L, Zeng XY, Wang BC (2019). Efficacy and safety of photodynamic therapy with amino-5-laevulinate nanoemulsion versus methyl-5-aminolaevulinate for actinic keratosis: a meta-analysis. Photodiagnosis Photodyn Ther..

[29] Morton CA, Dominicus R, Radny P (2018). A randomized, multinational, noninferiority, phase III trial to evaluate the safety and efficacy of BF-200 aminolaevulinic acid gel vs. methyl aminolaevulinate cream in the treatment of nonaggressive basal cell carcinoma with photodynamic therapy. Br J Dermatol..

[30] Reinhold U, Dirschka T, Ostendorf R (2016). A randomized, double-blind, phase III, multicentre study to evaluate the safety and efficacy of BF-200 ALA (Ameluz®) vs. placebo in the field-directed treatment of mild-to-moderate actinic keratosis with photodynamic therapy (PDT) when using the BF-RhodoLED® lamp. Br J Dermatol..

[31] Zhao S, Wu J, Wang C (2013). Intraoperative fluorescence-guided resection of high-grade malignant gliomas using 5-aminolevulinic acid-induced porphyrins: a systematic review and meta-analysis of prospective studies. PLoS One..

[32] Bickels J. Safety and efficacy study using 5-ALA oral administration as an adjuvant therapy on the rate of local tumor recurrence in patients who have desmoids tumors. Available at https://clinicaltrials.gov/ct2/show/NCT01898416. Accessed on Jan 6, 2020.

[33] Bickels J, Gortzak Y, Sternheim A, Kollender Y. 5-Aminolevulinic acid photoablation of fibrotic soft-tissue tumors. Presented at the 28th Annual European Musculoskeletal Oncology Society Meeting (Athens, Greece) April 29-May 1, 2015.

[34] Li Y, Zhou Q, Hu Z (2015). 5-Aminolevulinic acid-based sonodynamic therapy induces the apoptosis of osteosarcoma in mice. PLoS One..

[35] White B, Rossi V, Baugher PJ (2016). Aminolevulinic acid-mediated photodynamic therapy causes cell death in MG-63 human osteosarcoma cells. Photomed Laser Surg..

[36] Osaki T, Yokoe I, Sunden Y, et al. Efficacy of 5-aminolevulinic acid in photodynamic detection and photodynamic therapy in veterinary medicine. Cancers (Basel). 2019:11. 10.3390/cancers11040495 PMC6520946 30959982

[37] European Medicines Agency. Gliolan: EPAR - Scientific Discussion. Available at http://www.ema.europa.eu/ema/index.jsp?curl=pages/medicines/human/medicines/000744/human_med_000807.jsp&mid=WC0b01ac058001d124. Accessed on June 30, 2015.

[38] Perez MH, Rodriguez BL, Shintani TT, Watanabe K, Miyanari S, Harrigan RC (2013). 5-aminolevulinic acid (5-ALA): analysis of preclinical and safety literature. Food Nutr Sci.

[39] Teixidor P, Arraez MA, Villalba G (2016). Safety and efficacy of 5-aminolevulinic acid for high grade glioma in usual clinical practice: a prospective cohort study. PloS One..

[40] Chang NS (2016). Bubbling cell death: a hot air balloon released from the nucleus in the cold. Exp Biol Med (Maywood).

